# Case of Cancer of the Pancreas and Modified Transfusion of Blood

**Published:** 1874-01

**Authors:** Ephriam Cutter

**Affiliations:** Woburn, Mass.


### CASE OF CANCER OF THE PANCREAS AND MODIFIED TRANSFUSION OF BLOOD.

BY EPHRIAM CUTTER, M.D., WOBURN, MASS.

Mrs. W. B., aged twenty-four, was confined with her first child in August, 1872. She had a tedious labor, and was attended first by an uneducated midwife, and second by a not very learned homeopath. She did not have a good getting-up. Nursed her child on a bottle. She was afterwards attended by an eclectic, who said she had consumption and dosed, her thoroughly. A regular physician next attended her, who succeeded no better, and on March 16, 1873, my attention was called to the case.

She was confined to her bed on her back, and presented the most marked symptoms of anemia. She was white as a sheet; lips, alveolar processes, tongue soft and hard palates, tonsils, post-pharyngeal wall looked as if bleached with chlorine gas; face intelligent; eyes clear; tougue moist; pulse small, 120; respirations, 20; percussion normal; auscultation normal; heart's sounds unrythmical, feeble, not well pronounced. No bruit of anemia. There was some cough; bowels somewhat distended, clear on percussion; no tumor felt in abdomen; urine normal under microscope; no albumen; bowels rather regular; no dullness on percussion; hardness or enlargement over the left lower third (lateral) of thorax. There was vomiting and gastric distress constantly, with occasional remissions. Vomitus varied with the food. Fatty substances distressed her most and were soonest rejected. The skin of the whole body blanched deeply with a slight yellowish tinge. It was moist. The cutaneous veins contracted and almost obliterated.

Under the microscope (Tolles one-fifth objective, immersion without cover, B. eye-piece), the liquor sanguinis was found to be in large excess. The quantity of the white and red blood corpuscles diminished equably. That is, the normal relation of the red and white corpuscles was preserved, while the quantity was diminished, in other words, there was not an excess of the white blood corpuscles. The red corpuscles were the palest I ever saw.

The case was watched for a few days. Medicines to allay symptoms—chloral for sleep, wine and beef-tea injections with some mild preparation of iron were employed. At the expiration of this time the *diagnosis* was made from the following points:

(*a.*) That there was a grave blood disease involving the blood glands.

(*b.*) That the fact of the white corpusclcs not being in excess excluded the spleen. The absence of the usual signs of splenic engorgement favored this view.

(*c.*) That in the writer's opinion (derived from the researches of Dr. Salisbury, of Cleveland, Ohio), the pancreas is a blood gland next in importance to the spleen; hence the spleen being excluded, the pancreas would naturally be regarded as the offending organ.

(*d.*) That the pancreal was the offending organ, was evidenced by the fact that fatty substances were particularly offensive to the stomach, and were rejected undigested. Besides there was tenderness over the pancreal, but no bunch or tumor.

(*e.*) That the disease was organic and malignant, was covinced by tne grave character of the symptoms—their long continuance—their being not amenable to treatment. There was no cancer in the family. Mother died of phthisis.

From these data, the *diagnosis* was ventured of probable cancer of the pancreas. The case progressed from bad to worse—becoming insensible to chloral, opiates were not borne. The bowels were more and more distended; the heart palpitated more; More precordial distress; enemata rejected; urinary secretion normal; mind vacant and wandering; no sweat could be raised on the surface; strength rapidly failed, and death ensued on March 30, 1873. The following was the result of the autopsy, March 31, 1873.

*AUTOPSY.*

Lungs were healthy, except a few adhesions; heart also healthy. Stomach enormously enlarged; seventeen and a quarter inches long; at pyloric end, eleven inches in diameter; at other end, thirteen inches in diameter; it was distended with gas and grumous black fluid.

The spleen was natural size and healthy; the kidneys were enlarged and fatty; liver healthy. Pancreas or sweet-bread, affected with cancer of the schirrus variety, enlarged and hardened. Uterus and appendages normal.

The disease of the pancreal is very rare, and necessarily fatal.

In this connection, the writer would state that he was poisoned by the examination, on the inside of the distal phalanx of the left forefinger. Two days after, it became painful and swelled. The finger was wrapped with a bandage, and this bandage kept soaked with an almost caustic solution of iodine, which arrested the disease. The general symptoms were combated with twelve grains of cincho-quinine daily. But even after the symptoms abated, and I felt quite well, the finger was tender, angry and red. It discharged matter around the roots of the nail, which was loosened. The iodine did not arrest the diseased action.

Finding this was the case, a preparation was applied of lig. ferri persulphatis, U. S. P., and glycerin, equal parts. The effect was immediate. The pain was relieved, the pus stopped, the redness diminished, and the healthy restoration began.

This little therapeutical action is alluded to with more confidence, as this preparation is so highly esteemed by me in the topical treatment of throat diseases.

*MODIFIED TRANSFUSION OF BLOOD.*

Both my sympathies and energies being interested, I was anxious to try every possible expedient of relief. One day, mentioning transfusion, the patient eagerly caught at it and desired its performance. The experimental character of the expedient was fully set forth and made intelligible to both patient and friends. Their full consent was given. On Wednesday, the 26th of March, it was performed, as follows:

Taking the pneumatic aspirator, furnished by Codman & Shurtleff, of Boston, I attempted to pass one of its needles into one of the full distended veins of the patient's father—a stout, healthy farmer, of sixty years about. The needle enlarged the skin readily enough, but the elastic vein twisted away, allowing the needle to run along its side. Finding this method impracticable, the vein was laid bare by dissection, and the needle was readily entered into the vein, the blood running out of the minute canula. The barrel of the aspirator, warmed in water of a temperature of 100° Fahr., was then applied to the canula, and filled with the blood by suction with the piston. It was withdrawn, needle and all, and then plunged into the subcutaneous tissue of the right thigh of the patient, midway between the knee and the hip joint. The blood was then introduced beneath the skin.

The reason why it was not introduced into a vein was because of the smallness of the superficial veins of the skin; so introducing under the skin was the next best resort. (I have since learned that the smallness of the veins is no objection. It was only necessary to surround the arm with a ligature to expand the veins; dissect the median cephalic, and then inject.) The amount injected was about one ounce. Next day, the operation was repeated on the left thigh with about two ounces of blood.

The autopsy showed that the liquid portion was absorbed, leaving clots diffused through the areolar tissue, much as it is done in cases where there has been fracture of one of the long bones.

I have no doubt that had the patient lived, the whole would have been absorbed entirely. If, as Dr. Woodward says, there is migration of the white blood corpuscles out and in of the capillaries during healthy and physiological action, is it asking too much to suppose that this infused blood may not be absorbed into the circulation by these stomata first described by Cohnheim? It seems as if this subject deserved further consideration at the hands of the profession.

At any rate, this little experimentation of mine is offered as a small contribution in this direction. Whether valuable or not, it shows that in the case operated on, it did no harm, the patient dying from cancer.

